# Deconstructing Stem Cell Tumorigenicity: A Roadmap to Safe Regenerative Medicine

**DOI:** 10.1002/stem.37

**Published:** 2009-05

**Authors:** Paul S Knoepfler

**Affiliations:** a Department of Cell Biology and Human Anatomy & Stem Cell Program, University of California Davis School of MedicineSacramento, California, USA; b Institute of Pediatric Regenerative Medicine, Shriners Hospital For Children Northern CaliforniaSacramento, California, USA

**Keywords:** Stem cells, IPSC, hESC, Tumors, Regenerative medicine, Safety

## Abstract

Many of the earliest stem cell studies were conducted on cells isolated from tumors rather than from embryos. Of particular interest was research on embryonic carcinoma cells (EC), a type of stem cell derived from teratocarcinoma. The EC research laid the foundation for the later discovery of and subsequent work on embryonic stem cells (ESC). Both ESC isolated from the mouse (mESC) and then later from humans (hESC) shared not only pluripotency with their EC cousins, but also robust tumorigenicity as each readily form teratoma. Surprisingly, decades after the discovery of mESC, the question of what drives ESC to form tumors remains largely an open one. This gap in the field is particularly serious as stem cell tumorigenicity represents the key obstacle to the safe use of stem cell-based regenerative medicine therapies. Although some adult stem cell therapies appear to be safe, they have only a very narrow range of uses in human disease. Our understanding of the tumorigenicity of human induced pluripotent stem cells (IPSC), perhaps the most promising modality for future patient-specific regenerative medicine therapies, is rudimentary. However, IPSC are predicted to possess tumorigenic potential equal to or greater than that of ESC. Here, the links between pluripotency and tumorigenicity are explored. New methods for more accurately testing the tumorigenic potential of IPSC and of other stem cells applicable to regenerative medicine are proposed. Finally, the most promising emerging approaches for overcoming the challenges of stem cell tumorigenicity are highlighted.

## TUMORS, NOT EMBRYOS INITIATED THE DEVELOPMENT OF THE EMBRYONIC STEM CELLS FIELD

The link between stem and tumor cells in science is a very old one. The earliest research on highly pluripotent stem cells was conducted not on normal stem cells, but instead on embryonic carcinoma cells (EC) derived from teratocarcinoma. EC had unusual stem-like properties that attracted a great deal of attention at the time, including a surprising degree of plasticity. Teratocarcinoma and its benign cousin teratoma are the best examples of tumors with substantial populations of pluripotent stem cells as well as differentiated tissues. It may not be widely appreciated that when embryonic stem cells (ESC) were first isolated [1,2] from the mouse by two independent groups more than a decade after the discovery of EC, the existing knowledge base and reagents related to EC played pivotal roles. In fact, in one case ESC were established and cultured in EC conditioned media [2]. It was also noted that ESC expressed the same markers as EC. Thus, EC were the touchstone to which ESC were initially compared and the fact that ESC shared so many properties with established EC lines was argued as validation that ESC were pluripotent stem cells. ESC and EC could be differentiated into a myriad of cell types [3] and formed teratoma with many diverse tissues, suggesting the exciting conclusion that both these cell types had robust pluripotency (reviewed in [4]).

However, these studies also raised an important, still largely unanswered question. Why would ESC, supposedly normal counterparts to EC, also have the ability to cause tumors? The simplest but most troublesome answer is that ESC and EC are in fact, as was originally assumed, quite similar types of cells. Removing the inner cell mass (ICM) from the context of the early embryo and enforcing the culture of ICM cells in vitro produces a new cell type that does not normally exist in nature, that is, ESC. Although ESC mirror many of the normal, desired properties of the ICM cells such as near totipotency, they are not simply an in vitro manifestation of ICM cells but also almost certainly contain distinct properties, some of a more tumorigenic nature. The fact that the ESC field began with and in some ways depended on the much earlier discovery of EC highlights the intimate link between stem and tumor cells as well as between pluripotency and tumorigenicity.

The shift to a potentially more tumorigenic state as ICM cells transition to ESC in vitro may be driven by the method of creating ESC via selecting for those unique ICM cells that can be forced to grow in vitro. This selective process is predicted to go hand-in-hand with epigenetic changes that enable such growth. Thus, driving ICM cells to become ESC may be at the same time creating a cell type that is inescapably also pushed toward a tumorigenic phenotype. Just how different ESC are from ICM cells remains an intriguing question. It is important to note that even transplants of whole early embryos can drive teratoma formation as well so some of the forces driving teratoma formation are intrinsic to ICM cells and not because of their in vitro growth to produce ESC.

## THE TERATOMA ASSAY: BOTH A PLURIPOTENCY AND A TUMOR ASSAY

It is remarkable that, to date, one of the most common assays for demonstrating and studying the pluripotency of stem cells, including induced pluripotent stem cells (IPSC), is the teratoma assay. Often this is referred to as a pluripotency assay, but of course it is also a tumor assay. The fact that a key assay of “stemness” is also a tumor assay further illustrates the strong link between stem and tumor cells, a reality too rarely discussed in the field when interpreting results from teratoma assays. Even ignoring for the moment the ability of ESC and IPSC to produce malignant tumors in some cases [5,6], the production of benign teratoma as a side effect in humans given a hypothetical regenerative medicine therapy in the future, would be unacceptable. Such tumors could be numerous and would prove highly destructive to surrounding normal or regenerating tissue. Thus, a key concept is that stem cells, even those with potent self-renewal and pluripotency, will almost certainly never be directly used in regenerative medicine if they cannot be proven to lack the ability to cause teratoma in mice.

## WHAT IS THE MOLECULAR BASIS OF THE TUMORIGENICITY OF NORMAL STEM CELLS?

Recent studies indicate that many of the same master programming elements are at work in both stem cells and tumor cells [7–10]. This shared molecular machinery suggests that untangling the determinants of pluripotency from the programming responsible for tumorigenicity is going to be a major challenge. The links between pluripotency and tumorigenicity are exemplified by the fact that many of the genes used to produce IPSC are either outright established oncogenes such as Myc and KLF4 [11–13] or are in various ways linked to tumorigenesis such as Sox2 [14], Nanog [9], and Oct3 [15]. But perhaps no molecule embodies the interwoven nature of the pluripotency and tumorigenicity programs more than Myc itself. Overexpression of the Myc family of proto-oncogenes is linked with an array of human tumors and elevated Myc expression may have some role in all human cancer [16]. Not only is Myc expression itself shared between stem and tumor cells, but distinct groups of Myc regulated target genes are coexpressed in both malignant tumors and ESC [10].

Myc was one of the first oncogenes discovered and there are literally thousands of papers studying the function of excess Myc in cancer. However, it has only been more recently that the normal role of Myc in stem cell biology has been discerned. Loss of function models reducing or eliminating expression of Myc genes in stem cells consistently show disruption of the function of those stem cells [17,18] and c-Myc appears to be essential for normal STAT signaling in mouse ESC (mESC) required for self-renewal and pluripotency [19]. In terms of IPSC, although it is formally possible to create IPSC without Myc [20,21], the efficiency is dramatically reduced by the omission of Myc and the timeframe for appearance of colonies is greatly extended. In Yamanaka's groundbreaking IPSC paper, they reported being unable to make IPSC without Myc [22] further suggesting Myc strongly boosts IPSC formation. Thus, the stem cell field is faced with a catch-22 situation in that if one seeks to make stem cells safer by lowering Myc levels, a tandem reduction in the “stemness” of those cells may prove inevitable. The same appears to be true for other master stem cell regulators such as KLF4. Lowered levels of KLF family members including KLF4 substantially impaired ESC pluripotency and self-renewal, forcing ESC to differentiate [23].

## ARE PLURIPOTENCY AND TUMORIGENICITY COUPLED?

**Figure 1:**
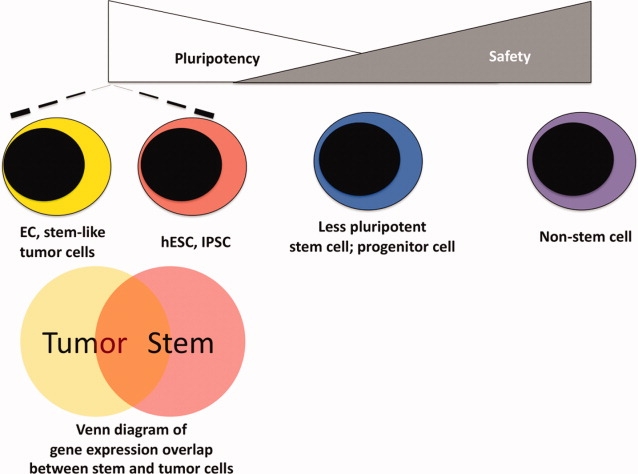
The relationship between pluripotency and tumorigenicity. Biological (top) and molecular (bottom) links between pluripotency and tumorigenicity are described. Abbreviations: EC, embryonic carcinoma cells; hESC, human embryonic stem cells; IPSC, induced pluripotent stem cells.

The dualistic natures of Myc and KLF4, linked to both tumorigenesis and normal stem cell biology, highlight the more general dilemma that the regenerative medicine field faces in trying to preserve self-renewal and pluripotency while eliminating tumorigenicity. A fundamental principle of cell biology may be that the greater the pluripotency and self-renewal properties that a stem cell possesses, invariably the higher the probability it will cause tumors (Fig. 1). Conversely, reducing the tumorigenic nature of stem cells may inevitably reduce the self-renewal and pluripotency of stem cells. Unfortunately, this means that one may not be able to completely eliminate the ability of a stem cell to cause tumors without robbing the cell of identity, its stem-like nature. In other words, to make a stem cell completely unable to cause tumors, you may have to make that cell no longer be a stem cell. However, not all stem cells even within the same culture have the ability to form tumors suggesting that pluripotency and tumorigenicity may not be so completely bound together. In this alternative model, “stemness” and tumorigenicity are highly related process, but separable and with important distinct molecular features.

## ANIMAL SAFETY MODELS: HOW TO DISTINGUISH BETWEEN LACK OF STEM CELL TUMORIGENICITY VERSUS HOST REJECTION?

Part of the challenge of resolving issues related to stem cell safety is that only few in vivo studies have been reported, particularly on human cells such as human ESC (hESC). Some studies with introduction of hESC into animal models have given apparently encouraging results. An example of such a study was one where rats were given a hESC transplant. They not only showed improvement of their Parkinson's disease symptoms, but at least for the 3 months of the study also did not develop detectable teratoma [24]. On the other hand, another study using an animal model system and stem cell transplant failed because of teratoma formation [25]. In all such studies, particularly with a “negative” result where no teratoma were detected, it is unclear whether the apparent lack of tumorigenesis is related to the inherent properties of the transplanted stem cells or rather reflects the level of immunosuppression in the animal model being used (i.e., a false negative). Lack of teratoma in animal models with stem cell transplants may most often be reflective of a failure of engraftment due to immune cells in the host killing the stem cells. Fortunately, new, improved humanized mouse model systems are continually being developed that may be more useful for assessing the tumorigenicity of stem cells [26].

Teratoma is not the only concern as hESC can also form malignant tumors. A recent study found robust malignant tumor-inducing capacity of hESC including H1 and HSF-6 [6]. IPSC can also form both teratoma as well as malignant tumors such as neuroblastoma and follicular carcinoma [5]. Thus, the potential risk to human patients from both teratoma and malignant tumors is quite real, yet remains difficult to estimate as no human trials of hESC or IPSC have been conducted at this time.

## PARALLELS BETWEEN INDUCED PLURIPOTENCY AND ONCOGENIC TRANSFORMATION IN THE FORM OF FOCUS FORMATION IN FIBROBLASTS

IPSC have been produced from a number of cell types, but most often from fibroblasts. The production of IPSC is eerily similar in a number of ways to a tumor-formation assay called “focus formation” in fibroblasts [27,28] with both able to be driven by Myc genes. Focus formation is an assay for testing tumorigenicity of specific genes in fibroblasts. In both focus formation assays and IPSC induction, monolayer cultures of fibroblasts are transduced with retroviruses, some of which encode oncogenes. The growth of cultures is continued at high density without passaging. Normal cells undergo contact inhibition and generally remain as a largely quiescent monolayer. In both IPSC and focus formation assays, the expectation is that at some point tightly packed growths of cells will form above the monolayer. In each case, these “colonies” exhibit escape from the normal quiescent state induced by contact inhibition. Both IPSC and oncogenic foci are transferable to form new cultures and both can cause tumors when injected into immunocompromised mice. The parallels between foci formation and IPSC production suggest these processes are perhaps related to some extent. It is quite possible that a fraction of colonies that form during IPSC induction are more similar to an oncogenic focus than an IPSC colony and may very well be essentially tumor colonies. If so, studies of these byproducts of the IPSC process may prove fruitful for furthering our understanding of the links as well as differences between stem and tumor cells.

## WILL NONGENETIC METHODS FOR PRODUCING IPSC REDUCE THE RISK OF TUMORS?

If one accepts the model that making a cell more stem-like predisposes that cell to cause a tumor, then IPSC are predicted to be inherently more tumorigenic than their nonstem cells of origin such as fibroblasts. However, even beyond this kind of modeling there are compelling reasons for worrying about IPSC tumorigenicity based on actual published data. Of greatest concern is that nearly all IPSC described in published works have been demonstrated to cause teratoma, proving pluripotency but also tumorigenicity, and that mice genetically derived to contain some tissues from IPSC have a malignant tumor incidence of 20% [29]. Genetic changes intrinsic to the IPSC generation process may pose risk of enhancing tumorigenesis through both the introduced genes themselves and in theory via the potential changes at specific integration sites.

The IPSC field is evolving rapidly and moving away from methods of induction that rely on genetic changes. This approach is in its early days with some very promising initial results [30,31], but predictions are that such a move generally should reduce tumorigenicity and improve safety. However, important questions remain. Will it ever be possible to make IPSC with absolutely no genetic changes? Can IPSC ever totally escape from dependence (whether via genetic or nongenetic approaches) on Myc, KLF4, and other possible oncogenes? Although it may appear that the IPSC field has already answered this affirmatively for Myc in that IPSC can be generated without added Myc, the omission of Myc reduces the efficiency of IPSC generation and yet these IPSC can still produce tumors in the form of teratoma [20,21,32]. These studies also do not address the role of endogenous Myc. In IPSC generated without genetic addition of Myc, the cells of origin may well be characterized by unusually high levels of endogenous Myc proteins required for the reprogramming and could make the cells prone to tumorigenesis.

## PROPOSING AN ALTERNATE ASSAY OF TUMORIGENICITY FOR IPSC AND OTHER STEM CELLS

To gauge the tumorigenic nature of IPSC most often researchers have used what will be termed the derived mouse assay. In this assay, through mouse genetics IPSC are used to contribute to the formation of chimeric mice in which some tissue lineages are derived from IPSC. The mice are then studied for tumor development. Using this assay, IPSC have been reported to cause malignant tumors in up to 20% of such derived mice [5], whereas IPSC generated without exogenous Myc have been reported to not form tumors [20,32]. The cause of the tumors has been thought to be most often due to reactivation of Myc from previously silenced viral insertions. The problems with the derived mouse assay are twofold. First, the IPSC must go through early embryogenesis and are subject to the powerful embryonic reprogramming forces that are predicted to dramatically reduce the apparent tumorigenicity of the cells. Second, the derived mouse system for studying IPSC tumorigenicity bears no resemblance to how cells would be used in regenerative medicine where they would either be focally injected in a site to be repaired or administered intravenously (IV). Both focal injection and IV administration of IPSC as a means for studying IPSC tumorigenicity have not been reported in the literature. Unfortunately, these assays are expected to show higher rates of tumorigenicity than the derived mouse assay, but more accurately reflect risk to patients. Another problem with the derived mouse assay is that currently it is unusable for studying tumorigenicity of human IPSC. Researchers appear to be disinclined to put human IPSC into mouse embryos, perhaps due to the ban on putting hESC into a mouse embryo and taking it beyond day 8 of growth. Thus, other than their robust teratoma inducing abilities, the reality is that the stem cell field knows almost nothing about the tumorigenicity of mouse IPSC and essentially nothing about that of human IPSC in a context relevant to regenerative medicine.

## “THESE STEM CELLS LOOK TOO GOOD TO BE NORMAL”: COMMON PRACTICES WORSEN THE INHERENT TUMORIGENICITY OF STEM CELLS

Although stem cells have an intrinsic predisposition to causing tumors under certain conditions, this predilection can easily but unknowingly be enhanced by how the stem cells are grown by researchers. The simple act of removing stem cells from organisms and growing them in culture profoundly changes the biology of the cells in ways that makes them more tumorigenic. Although the hematopoietic stem cells in bone marrow can be in essence directly transplanted to patients without loss of repopulating activity, in most cases the in vitro culture of stem cells is an unavoidable step. Further, the rate of a surprisingly large number of genomic alterations (reviewed in [33]) increases with time in culture and is at least one general mechanism of enhanced tumorigenicity [34]. The karyotypic alterations in cultured hESC have been proposed to mirror tumorigenic events that occur in vivo [33] and to at least in part contribute to tumorigenicity through enhanced proliferation. In general, the tumorigenicity of stem cells is predicted to increase proportionately with the length of time that they are cultured. An exception to this is mesenchymal stem cells (MSC), which appear to be culturable for weeks without clear adverse consequences. A long-term study MSC found a very positive biosafety profile [35] and MSC are currently in clinical trials with encouraging results (reviewed in [36]). Thus, more generally stem cells other than IPSC and ESC may prove more amenable to culturing, but problems such as karyotypic changes can also occur in these other stem cells indicating that minimizing culture time should always be a key objective.

The “best” ESC are often considered to be those that grow fairly rapidly, form tightly packed colonies, have a low rate of spontaneous differentiation, and are readily passageable. As researchers grow ESC, there may be a tendency to selectively propagate colonies and even work with ESC lines that more generally fit the above criteria. Such ESC are almost certain to be endowed with specific molecular characteristics that make them easier to grow, but at the same time the traits described earlier, when selected for, may tend over time to enrich for stem cells that have enhanced tumorigenicity. Interestingly, a recent study indicates that hESC cultures are heterogenous, containing cells with widely varying tumorigenicity and some variant sublines were isolated with enhanced tumorigenicity [37]. Although this complicates the picture of hESC-based regenerative medicine, it also suggests an opportunity for isolating variants that possess low or absent tumorigenicity. Paradoxically, the best ESC for regenerative medicine may be the hardest to propagate because they exhibit the following properties: slow growth, a high rate of spontaneous differentiation, and low colony forming potential. Thus, the safest ESC for regenerative medicine therapies are predicted to be those that are the most difficult to culture and the easiest to culture may be the least safe.

## AN UNANTICIPATED RISK FROM INDUCED EPIGENETIC CHANGES?

Mostly under the radar in the field of stem cell safety are potential undesirable side effects of epigenetic changes in IPSC and hESC that are undetectable by karyotyping, but could have profound effects on cell biology as the biology of normal hESC is regulated by epigenetic programming [38]. Epigenetic changes are also postulated to play a key role in the reprogramming at the heart of IPSC formation [11,12] and are modeled to be instrumental in creating IPSC both with or without genetic changes, particularly in the latter case. Thus, it is critically important to more fully study the global epigenetic changes associated with pluripotency and especially induced pluripotency, even if IPSC are ultimately produced without genetic changes. Epigenetic alterations may in part confound the efficacy of moving away from genetic changes through promoting tumorigenesis themselves. Such changes also occur during establishment and passaging of hESC [39], whose epigenome is highly unstable [40], almost certainly enhancing tumorigenicity. Characterizing the relationships among the epigenome, pluripotency, and tumorigenicity should prove of great benefit for developing safe regenerative medicine.

## APPROACHES TO SAFE STEM CELL-BASED REGENERATIVE MEDICINE THERAPIES

**Figure 2:**
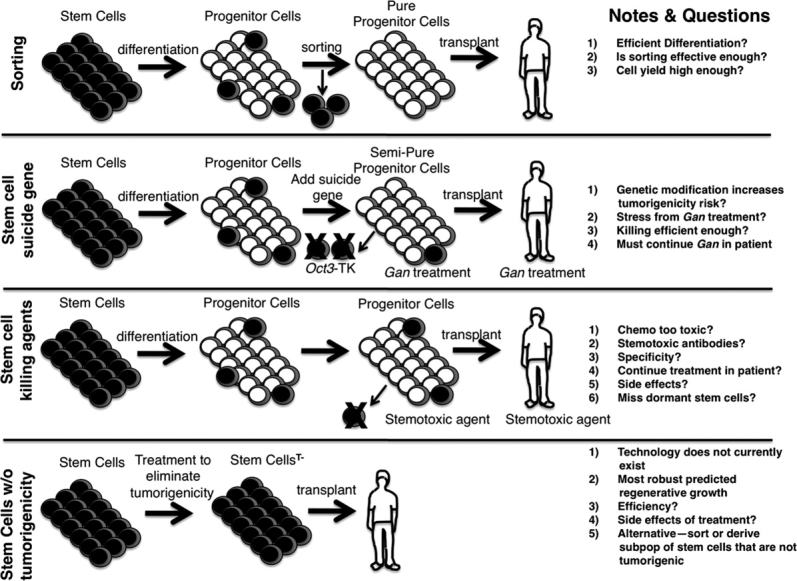
Methods to address stem cell tumorigenicity to develop safe regenerative medicine therapies. Four possible approaches are outlined. “Stemotoxic” refers to agents or methods that are specifically toxic to and hence kill stem cells.

The four most promising approaches to make stem cell-based regenerative medicine safer are discussed here (Fig. 2). Some pretransplant screening is applicable to all four approaches. Safety screening could encompass everything from assays of genome integrity (chromosome number, deletions, and duplications) to gene expression array profiling as well as micro RNA patterns and perhaps even epigenetic screening. These approaches should enhance the safety of any regenerative medicine therapy.

### Transplants of Progenitors

In a general sense, the reason that relatively “normal” stem cells such as ESC can in turn still cause tumors is because they are programmed to be robust tissue and organ growers. Tumors are abnormal organs or tissues, which a growing body of work suggests in many cases, have developed from and perhaps are maintained via their own population of stem cells. The notion that a wide variety of tumors beyond those such as teratocarcinoma and teratoma may contain stem cells is gaining widespread acceptance (reviewed in [41]). These tumor or cancer stem cells, also termed tumor-initiating cells, seem to share many traits with normal stem cells, but are predicted to have at least partially impaired pluripotency. In that sense, tumor stem cells may be akin to racecars with accelerators (indefinite self-renewal potential) but bad brakes (differentiation potential/pluripotency).

The simplest way to slow or even eliminate the tumorigenicity of normal stem cells prior to transplantation may be to take advantage of their natural “brakes” or pluripotency by partially differentiating them into progenitors. Therefore, a promising proposed method for making stem cell-based regenerative medicine therapies safer may seem paradoxical: to not transplant stem cells at all into patients. This avenue has gained wide acceptance as the most promising approach to regenerative medicine. The idea is to use the stem cells to produce progenitor or precursor cells of the desired lineage and then transplant progenitors purified by sorting (Fig. 2). On January 23, 2009 such an approach was given approval by the Food and Drug Administration based on hESC-derived oligodendrocytes. With sufficient purity, weeding out through differentiation coupled with sorting all or nearly all contaminating stem cells that remain, the progenitor transplant should be both safe and effective. The sorting could be either positive (sorting for the progenitors based on markers) or negative (sorting via stem cell markers for their elimination). Because differentiation is a dynamic process, not an “on/off” switch, there will always be residual stem cells remaining in differentiated cultures and sorting is not perfect. Thus, the most practical approach to safe regenerative medicine would be some combination of differentiation, sorting, and one of the two general approaches outlined below (first two subsections) to kill residual stem cells.

It remains unknown just how pure the progenitors must be to be safe. How few remaining stem cells are enough to cause concern about tumors? If the answer is “zero,” then it may be difficult to achieve this goal because the reality is that the only pure cell population is that consisting of a single cell. It seems much more probable that the presence of a few stem cells may not pose a serious risk. However, the remaining undifferentiated stem cells may be unique and have a much higher risk of generating a tumor because they in some manner escaped being differentiated, perhaps reflecting a partial impairment of pluripotency that brings them one step closer to a tumorigenic phenotype. The few published studies addressing this question leave the issue largely unresolved. For example, a study of use of hESC for Parkinsonian rats found that differentiation of the cells prior to transplant lowered the incidence of teratoma [42]. However, high levels of natural killer (NK) cells in many rat and mouse animal models appear to kill most or all injected stem cells, questioning the validity of such safety studies when results are negative. Further complicating the story is the observation that ESC are in fact more susceptible to killing by NK cells than are differentiated ESC due to differences in cell surface proteins [43].

### Introduction of a Stem Cell Specific Suicide Gene

The stable genetic introduction of a suicide gene such as thymidine kinase (tk) into stem cells has been reported to be effective in combination with Ganciclovir (*Gan*) treatment [44]. However, in this study, the treatment was not stem cell specific and would have also killed any differentiated progeny from those stem cells in a hypothetical treatment situation causing it to fail. Differentiated teratoma cells were also readily killed by *Gan* treatment. Nonetheless, relatively simple modifications, such as using *oct3* or *nanog* promoter driven expression of tk, would make the system more stem cell specific to ideally kill only those hESC that have escaped differentiation. Of concern is the fact that it remains unknown if all hESC express what are thought of as the key stem cell factors such as *oct3* and *nanog*. Although populations of hESC do express these seemingly without exception, it is unclear whether small, but functionally relevant subpopulations may not. Another open question is whether transplantation of hESC and engraftment of hESC in the host could lower or shut off expression of suicide genes driven by stem cell promoters either immediately following transplant or at a much later date. Even with efficient stem cell killing, the possibility of patients requiring life-long treatment with Gan or other agents to suppress growth of residual stem cells raises the issue of possible reemergence of proliferating, drug-resistant stem cells possibly in turn leading to tumors at later dates.

The major concern with the suicide gene approach is its requirement for genetically modifying the stem cells, which could raise the risk of tumorigenicity from the beginning. However, a recent study of the safety of viral transduction of human hematopoietic stem cells and MSCs in which animals were followed for up to 18 months found no evidence of tumorigenesis, suggesting that limited genetic modification of the type needed to introduce a single suicide gene may be safe [35]. If clonal hESC derivatives can be produced, viral integration sites can be mapped to ensure that they are at the very least in noncoding genomic locations and ideally at a large distance from genes, further enhancing safety. Similarly, it is possible that viral vectors can be designed to integrate with a high frequency at specific sites at regions distant from genes.

### Directed Killing of Residual Stem Cells Based on a Nongenetic Method

If the safety of stem cells with introduced suicide genes turns out to be a serious obstacle, other approaches for weeding out residual stem cells may be needed. Although it is formally possible that patients receiving a stem cell-based regenerative medicine therapy could be treated with a broad-spectrum chemotherapeutic agent (chemo) postdifferentiation, for many already ill patients such treatments may be too toxic and it is unclear how effective they would be at killing residual stem cells, particularly if they were temporarily dormant. Although hESC and IPSC are rapidly growing cells that should in theory be killed by chemo, residual stem cells from an hESC or IPSC transplant may very well take on a quiescent, chemoresistant character in vivo. Thus, much more specific killing of residual stem cells is desirable. The most promising approach to this end is to use killer antibodies directed against antigens present on the surface of hESC such as SSEA-4 or a member of the TRA family. New hESC surface antigens are currently being discovered and tested such as podocalyxin-like protein-1, which appears quite promising as a cytotoxic agent specifically against undifferentiated hESC [45]. One major concern with this “stemotoxic” approach is how specific these methods would be at targeting stem cells as opposed to other cell types. Also, would the antistem cell treatments have to be continued in the patient and with what side effects? What would be the consequences if these treatments killed a significant number of endogenous stem cells in specific organs in the patient? At this time too little is known about the possible expression of the targeted antigens in adult stem cells and it remains a serious concern.

### Use Stem Cells Themselves for transplant, but First Eliminate Tumor Forming Potential Without Genetic Modification

In theory, the simplest approach to regenerative medicine and the one expected to lead to robust regenerative tissue growth would seem to be to use stem cells themselves, but ones that had been treated in such a way that they were no longer tumorigenic. Of course, at this time no such methodology exists and the notion of using stem cells directly for transplants would appear to be strongly out of favor with regulators due to the robust ability of hESC to form teratoma [46].

A similar conceptual kind of approach, selective purging of malignant cells from bone marrow leading to enhance safe transplantation of hematopoietic stem cells, has not consistently been effective (reviewed in [47]), but that may be a much taller order since malignant cells are already abundant. There remains hope more generally that mixed populations of tumorigenic and nontumorigenic stem cells may be separable or that the tumorigenic subpopulation may be selectively targetable. Further, as mapping continues of the molecular mechanisms by which stem and tumor cells are programmed, differences will continue to be revealed and those unique traits may pave the way for approaches to eliminate tumorigenicity while preserving pluripotency.

## FUTURE DIRECTIONS

The road from where we are today to a future with IPSC- and hESC-based regenerative medicine therapies being safe and more common treatment modalities is not a clear, linear one. However, basic and translational studies into the tumorigenic nature of stem cells are going to be collectively an essential bridge to cross along the way. Further advances in our understanding of tumor stem cells and tumorigenesis more generally will also provide additional fuel for these advances. Finally, a much more open discussion and investigation of the tumorigenic nature of stem cells than has yet to occur, particularly that of IPSC and hESC, will undoubtedly prove essential for the development of safe and effective regenerative medicine therapies.

## DISCLOSURE OF POTENTIAL CONFLICTS OF INTEREST

The author indicates no potential conflict of interest.
